# Employees’ emotional awareness as an antecedent of organizational commitment—The mediating role of affective commitment to the leader

**DOI:** 10.3389/fpsyg.2022.945304

**Published:** 2022-07-29

**Authors:** Marisa Santana-Martins, José Luís Nascimento, Maria Isabel Sánchez-Hernández

**Affiliations:** ^1^Business Administration and Sociology Department, School of Economics Sciences and Management, University of Extremadura, Badajoz, Spain; ^2^Centro de Administração e Politicas Públicas, Intituto Superior de Ciencias Sociais e Politicas, Universidade de Lisboa, Lisbon, Portugal

**Keywords:** organizational commitment, commitment to the leader, employees´ emotional awareness, dual commitment, workplace commitments

## Abstract

Commitment has been perceived as a strategic topic in organizations due to its positive effect on retaining talent, increasing performance, or boosting employees’ innovative behavior. However there are many focis of commitment in the workplace, which has represented a challenge to human resources management, who need implement measures to improve the employee’s commitment. Recent research has suggested a need to conduct studies about commitment, namely antecedents and the relationship between different focis, to understand the dynamic and directionality between them. Hence, the purpose of this work is to analyze how employees’ emotional awareness relates with two focis of commitment (the leader and the organization), also assessing the mediating role of affective commitment to the leader. The study uses structural equation modeling and Lisrel to test the hypotheses considering the multidimensionality of organizational commitment (affective; normative; and continuance), employees emotional awareness (understanding self-emotions; self-control when facing criticism; and understanding others’ emotions), and the affective commitment to the leader, under the scope of Social Exchange Theory. The Mackinon’s *Z* Test was used to assess the mediation role of affective commitment to the leader. The sample is composed for 403 employees from two multinational companies. The results provide empirical evidence about the mediating role of affective commitment to the leader in the relationship between employees’ emotional awareness and organizational commitment, and the employees’ emotional awareness as an antecedent of commitment. The implications for theory and practice are discussed.

## Introduction

Commitment has been increasingly considered a significant topic for organizations. Strategic interest in workplace commitment is related to the positive effects that it can have on employees, which are reflected in better organizational outcomes (Meyer, 2009; Beer et al., 2015; Markoulli et al., 2017; Culibrk et al., 2018; Klein et al., 2020; Lee et al., 2020).

Several studies have suggested a positive influence of commitment on employees’ motivational levels, which leads to increased levels of performance and innovation, while turnover and absenteeism rates that can harm business results also decrease (Battistelli et al., 2013; Xerri and Brunetto, 2013; Meyer, 2016; Lapointe and Vandenberghe, 2017; Zhang et al., 2018; Bak, 2020). The studies also reveal that the positive effects of commitment are also observed at the level of employees’ wellbeing, where increased prosocial behaviors in the organization lead to people-oriented organizational culture.

It can be said that the development of commitment measures in the workplace helps organizational sustainability (Murray and Holmes, 2021).

This interest and the need to undertake studies that enable a better understanding of commitment have come under attention over the last few decades. However, this need has intensified considerably in light of the changes we have witnessed regarding work relationships and how this influences how employees currently commit themselves to their workplace (Morrow, 2011; Hansen and Leuty, 2012; Heaphy et al., 2018).

This context has posed challenges to managing commitment, especially with regard to planning human resource policies that have an effective impact (Beer et al., 2015). The current difficulty in managing commitment stems not only from the change in paradigms associated with the new generations, but also from the need to better understand the multiplicity of focis for commitment that co-exist in the workplace.

Organizational commitment has been a target which has come under much study in recent years. However, research suggests that the strength of employee commitment to the organization has remained relatively stable over the last three decades. This, underline the probability of other focis of commitment gaining more relevance, such as commitment to the leader, colleagues, among others (Meyer et al., 2015; Meyer, 2016; van Rossenberg et al., 2018, 2022; Eisenberger et al., 2019; Klein et al., 2020).

Studies on commitment to the leader, whether conceptual or empirical, are relatively recent and remain scarce, despite this being a factor with a crucial effect on promoting and implementing organizational transformations, as it has a direct impact on employees (Jin and McDonald, 2017; Lapointe and Vandenberghe, 2017; Benevene et al., 2018; Bak, 2020). The role of leadership has a significant impact on organizational culture, promoting the expected values and behaviors. According with Saeed et al. (2022) ethical leadership has a great influence on the followers´ knowledge sharing, what is crucial to a culture of countinuous improvement because it can influence employees development and performance, and also the quality of services. According with these authors, employees’s professional commitment plays a moderating role on this behavior. In that sense, the developments of studies with different focis of commitment, and its relation with leadership, also underline the need to investigate the leader as a focus of commitment.

Likewise, there is unanimous recognition in the community of researchers that it there is a need to learn more about commitment, to more thoroughly explore its different antecedents and how they influence the processes of commitment, combining studies with different focis and exploring it multidimensionality (van Rossenberg et al., 2022).

Since the commitment process is inherent to the individual’s perceptual assessment, this study seeks to identify the extent to which employees’ emotional awareness can be considered an antecedent of organizational commitment and affective commitment to the leader, under the scope of the Social Enhance Theory (SET; Homans, 1958) and Affective Events Theory (AET; Weiss and Cropanzano, 1996). The emotional awareness brings to the employees the ability to identify and manage emotions according with the context, being able to identify not only their own emotions, but also those of others, and adapt their behavior appropriately (Côté, 2014; Helvac and Yilmaz, 2020).

Thus, to contribute to fill this gap, supporting on AET the aims of the present study are twofold. The first goal is to analyze the extent to which employees’ emotional awareness can be considered an antecedent of organizational commitment and affective commitment to the leader. Complementary, and based on SET, the second goal is to explain the leader member affective exchange. In other words, it is to analyze the extent to which affective commitment to the leader is a mediator of the process of employees’ commitment to the organization. Finally, the discussion of the results of our study will provide some implications for theory and practice.

## Theoretical framework and hypotheses development

### Employees’ emotional awareness as an antecedent of organizational commitment

Commitment is seen as a connection between an individual and a target; when it depends on extrinsic and intrinsic aspects of the individual, it can lead to behavioral stability (Meyer and Herscovitch, 2001).

One of the most quoted conceptualizations of commitment is the three-component model (TCM) put forward by Meyer and Allen (1991). This model, which has been revisited in various studies, is composed of the affective, normative, and continuance dimensions. From the perspective of organizational commitment, within the affective dimension, individuals create an affective and emotional bond with the organization and stay because they like it, and identify with it. The normative dimension implies a duty of moral obligation, and a feeling of indebtedness to the organization. Within the continuance dimension, individuals commit based on factors of an instrumental nature (material or monetary), which generate costs associated with change (Becker, 1960; Allen and Meyer, 1996; Meyer and Allen, 1997; Powell and Meyer, 2004; Klein et al., 2009).

Recent models suggest that commitment is influenced by antecedent variables that influence commitment processes according to various commitment focis. These variables can be of close influence (e.g., the nature of the task; relationships, and the status held in the organization) and of distant influence (e.g., personal characteristics, management practices, organizational climate, and culture; Meyer and Allen, 1997; Meyer and Herscovitch, 2001; Meyer, 2014; Klein et al., 2020). Thus, as commitment is a psychological state which is based on a set of perceptual assessments, the dynamics inherent in the way individuals perform these assessments will influence their behavioral process toward one or more focis (Meyer and Allen, 1997).

In view of this, the emotional response of an individual is closely related to their degree of awareness and their ability to interpret the facts they experience. How individuals interpret emotions, cognitive dynamics, and physical sensations determines their behavior and actions. There are several studies focused on positive emotions in the leader–member exchange literature (Cropanzano et al., 2017; Herman et al., 2018). In fact, social exchanges in organizations are the basis of the two-way relationship between leaders and employees (Bishop et al., 2005; Bhal et al., 2009).

Thus, the emotional experience has an implicit physical and intellectual impact, which triggers an emotional state affecting the individual’s experience and interpretation in a given situation and context.

When the emotional process is carried out with a greater degree of awareness, it implies a more constructive emotional response, as it enables the individual to re-evaluate both the specific situation and the way in which they react emotionally (Smith and Lane, 2015; Panksepp et al., 2017; Smith et al., 2018).

For this current study were considered three variables that measure individuals’ emotional awareness, such as Understanding self-emotions (USE); Self-control when facing criticism (SFC); and Understanding others’ emotions (UOE), based on the model developed by Rego and Fernandes (2005), previously adapted from the emotional intelligence model of Mayer and Salovey (1997). In short, employees’ emotional awareness can positively influence their day-to-day experience in the organization as well as their interpersonal relationships, and therefore also positively influence their organizational commitment. Thus, following this assumption, the first general hypothesis is formulated:

Hypothesis 1: Employees’ emotional awareness is positively related to organizational commitment.

According to Rego and Fernandes (2005), understanding self-emotions refers to the way in which individuals interpret their emotions and the event that triggered the emotion. This analysis allows individuals to understand what they feel and why enables them to regulate emotion constructively. This intellectual process can lead to individuals having greater awareness regarding the aspects with which they empathize and identify, and also positively influence affective, normative, and continuance organizational commitment. In view of the above, the following specific hypothesis is formulated:

*Hypothesis 1a*: Understanding self-emotions is positively related to organizational commitment.

Self-control when facing criticism refers to individuals’ ability to recognize and control their emotions in situations where they are the target of criticism. It implies the individual’s ability to understand the reason for the criticism and, consequently, to know how to deal with it (Rego and Fernandes, 2005). As mentioned by Smith et al. (2018), when employees are criticized, they can interpret this context in several ways. Individuals with greater emotional awareness will tend to interpret criticism constructively, drawing on it to self-correct and be aligned with what is intended. On the other hand, a lesser capacity for emotional management implies that individuals interpret feedback only as criticism; this may lead to fear of losing one’s job, or to becoming insecure because they think that they are not able to live up to expectations. Thus, the following hypothesis is formulated:

*Hypothesis 1b*: Self-control when facing criticism is positively related to organizational commitment.

Understanding others’ emotions encompasses individuals’ ability to identify and understand the emotions of those with whom they interact and adapt their interaction according to this interpretation. This intellectual dynamic leads individuals to regulate their behavior and communication to be in keeping with the context and the interlocutor (Rego and Fernandes, 2005). The quality of the interaction provides a greater ability to relate positively to the different stakeholders, with a certain level of emotional connection; this can promote the development of affective bonds and a sense of duty toward the organization. On the other hand, this capacity for emotional management can also provide individuals with the construction of solid relationships that foster continuity and the construction of a career in the organization. In view of the above, the following hypothesis is formulated:

*Hypothesis 1c*: Understanding others’ emotions is positively related to organizational commitment.

### Employees’ emotional awareness and affective commitment to the leader

Although organizational commitment is one of the most studied constructs, the same is not the case for commitment to a leader; this target of commitment is yet to be fully explored (Meyer et al., 2015; Becker, 2016; Klein et al., 2020; van Rossenberg et al., 2022). Leaders play a key role in promoting and implementing organizational transformations, as well as in day-to-day management and teams. As a figure who actively and continuously intervenes, the leader directly impacts employees’ experience in the organization (Bycio et al., 1995; Avolio et al., 2004; Eisenberger et al., 2010). Some studies suggest that leadership style influences employees’ organizational commitment (Bass et al., 2006; Zheng et al., 2015; Benevene et al., 2018; Zhang et al., 2018; Bak, 2020). However, the leader as a commitment target has only been approached relatively recently, and is still the subject of few studies, whether conceptual or empirical (Stinglhamber and Vandenberghe, 2003; Becker et al., 2009; Meyer, 2009; Strauss et al., 2009; Meyer et al., 2015; Klein et al., 2020; van Rossenberg et al., 2022).

Despite the multidimensional nature of commitment, authors such as Klein et al. (2014) and Meyer et al. (2015) argue that commitment to a leader tends to be a unidimensional construct, where the affective dimension has the greatest consistency, and where the normative and continuance dimensions are strongly correlated.

There is unanimous agreement that leaders’ actions influence employees, but the extent to which employees’ emotional awareness influences the way they interpret actions and interactions is not clear, as well as the role they play in the commitment process. The AFC argues that emotions are a significant part of human beings, from with they support substantially their actions and reactions. Therefore, emotions have a great impact in organizations, in the relationships between the stakeholders, as well as on commitment in the workplace. Emotions are internal events that occur within an actor as a result of social exchange emerging when two or more people exange valued outcomes such as rewards or payoffs (Lawler and Thye, 2006). The study of emotions and affective experiences in organizations is not new (Fredrickson, 2000; Barsade and Gibson, 2007). Treating emotional awareness as central feature of social exchange the common knowledge will be updated and enriched, through the social sharing of emotions at work.

The interpretation of emotions by the individual requires an assessment of the situation they are experiencing, and they will thus create representations or will rely on representations which were previously created in similar situations (Smith et al., 2018). This analysis can be conscious or not, where the assessment carried out is based on: (i) whether the situation is new or familiar; (ii) whether or not it is relevant to the current concerns of the individual; (iii) whether or not it is congruent with their objectives; (iv) whether it is within or outside their control; and (v) whether or not it is consistent with their norms or values (Brosch and Sander, 2013; Smith et al., 2018). A study of Zia et al. (2018) presented empirical evidences that employees´ emotional intelligence has a positive influence during conflict resolution strategies by supervisors, and also contribute to organizational citizen behavior among the group members. It is therefore assumed that, by providing a greater ability to analyze and manage emotions, both for individuals and for those with whom they interact, employees’ emotional awareness leads to a more constructive and healthy leader–member exchange. In view of the above, the following general hypothesis is formulated:

*Hypothesis 2*: Employees’ emotional awereness is positively related to affective commitment to the leader.

Emotional awareness supports individuals by fostering a greater ability to face everyday situations in a satisfactory manner. Faced with the challenges that arise, they use these experiences to structure their own development (Yip and Côté, 2013; Côté, 2014; Rimé, 2015; Smith et al., 2018). Understanding self-emotions is expected to enable the individual to be aware of the affective connection to the leader, contributing to an increasing awareness of the affective bond of commitment. Thus, the following specific hypothesis is formulated:

*Hypothesis 2a*: Understanding self-emotions is positively related to affective commitment to the leader.

Leader–member exchanges are imbued with moments of positive and negative feedback, in which employees’ emotional awareness can represent an important aspect of managing feedback. Self-control when facing criticism assumes that employees take feedback as constructive and as an integral and fundamental part of their development and alignment with objectives. Thus, Self-control when facing criticism is expected to positively influence affective commitment to the leader, with feedback being viewed as a guide to their development, and with the employee feeling grateful for having it. In view of the above, the following specific hypothesis is formulated:

*Hypothesis 2b*: Self-control when facing criticism is positively related to affective commitment to the leader.

Understanding others’ emotions allows employees to identify and align their behavior with the emotions of those with whom they interact. Cost–benefit analysis plays a major role in the social exchange process at work according to the SET (Homans, 1958). This theory is one of the most relevant frameworks in organizational behavior at the moment in different disciplines (Cropanzano and Mitchell, 2005). Under this paradigm, employees essentially take the benefits of the relationship with the leader, and with the organization, and subtract the costs to determine how much it is worth. It is important to highlight that this form of interaction driven by the individual interest of the employees is likely to transform into collective emotions (Lawler et al., 2014) positively contributing to organizational culture (Rimé, 2020).

According with Zia et al. (2018) in conflict contexts the employees emotional intelligence allows to a better understanding of leader’s conflicts resolution strategies, impacting also the employee’s behaviors on the organization. In that sense, the ability to understand other’s emotions permits to the individual manage their actions and build positive relationships in the workplace, what enables the process of affective commitment to the leader. In this way, the following specific hypothesis is formulated:

*Hypothesis 2c*: Understanding others’ emotions is positively related to affective commitment to the leader.

### The relationship between affective commitment to the leader and organizational commitment

Although there is consensus around the existence of multiple focis of commitment in the workplace, the same is not the case regarding their relationships and directionality, and few studies have undertaken an approach to two or more commitment focis (Klein, 2013; Meyer et al., 2015; Becker, 2016; Klein et al., 2020; van Rossenberg et al., 2022).

A recent study by Meyer et al. (2015), as an extension of the study by Stinglhamber and Vandenberghe (2003) which focused on two commitment focis, namely the organization and the supervisor, suggests that conceptual and empirical research on supervisor commitment is necessary, as well as its relationship with organizational commitment.

According to the aforementioned, the leader is one of the main actors in the organization, with active responsibility for the success and implementation of organizational measures. As such, it is the leader who interacts with employees and has a direct effect on individuals in the daily management of their responsibilities (Stinglhamber et al., 2015; Lapointe and Vandenberghe, 2017; Wu and Parker, 2017; Zhang et al., 2018). In this process of management and interaction, the leader can be an important target of commitment. Some studies have shown that the leadership style, as well as the way leaders give feedback to their teams, and the perception of interpersonal justice, can influence organizational commitment (Lapointe and Vandenberghe, 2017; Tetteh et al., 2019; Bak, 2020).

From the perspective of the dark side of leadership, Nadeem et al. (2020) identified the negative influence of destructive leadership on workplace and personal deviance, where emotional exhaustion plays a mediation role in the relationship. In this study is clear that the leadership can be oriented to create organizational damages, and even influence employees to act accordingly with this kind of interests. In this case, the employees´ emotional exhaustion conduct to workplace deviance and interpersonal deviance.

In contrast, positive leader behaviors influence many positive aspects, as Zada et al. (2022) have argued; servant leadership behavior promotes knowledge sharing, but also brings to the relationship some kind of proximity that supports psychological safety at work, where cooperative behaviors are common while discouraging immoral behaviors. The same indicates the study of Fatima et al. (2017) where participative leadership influences employee’s commitment to change, and increases their innovative work behavior. Once again, the leader plays a relevant role in commitment in the workplace, reinforcing the need to expand studies about the leader as foci of commitment, and its relationship on organizational commitment.

The study by Eisenberger et al. (2010) also suggest that the way employees perceive the leader’s organizational embodiment positively influences their organizational commitment, as the leader is seen as a representative of the organization. The emotional awareness brings to the employees the ability to identify and manage emotions according to the context, being able to identify not only their own emotions, but also the others´ emotions, and adapt their behavior appropriately. Similarly, Stinglhamber et al. (2015) presented empirical shreds of evidence about the influence of transformational leadership on follower’s affective organizational commitment.

Based on these studies, affective commitment with the leader is expected to lead to affective organizational commitment, insofar as the affective bonds developed are directed toward a figure that represents the organization. Therefore, the quality of the leader–member exchange which maintains a satisfactory and close relationship may lead to the development of a sense of duty toward the organization, positively influencing normative–organizational commitment. Leaders have increasingly assumed an important role in the career development of their team members, establishing a relationship where the feedback is an important key for improvement (Crawshaw and Game, 2015; Bak, 2020). Therefore, this context stimulates the affective commitment to the leader, having inherent career interests, so a positive influence of affective commitment with the leader is expected in the continuance of organizational commitment. Thus, the following general hypothesis was formulated:

*Hypothesis 3*: Affective commitment to the leader is positively related to organizational commitment.

**Figure 1:**
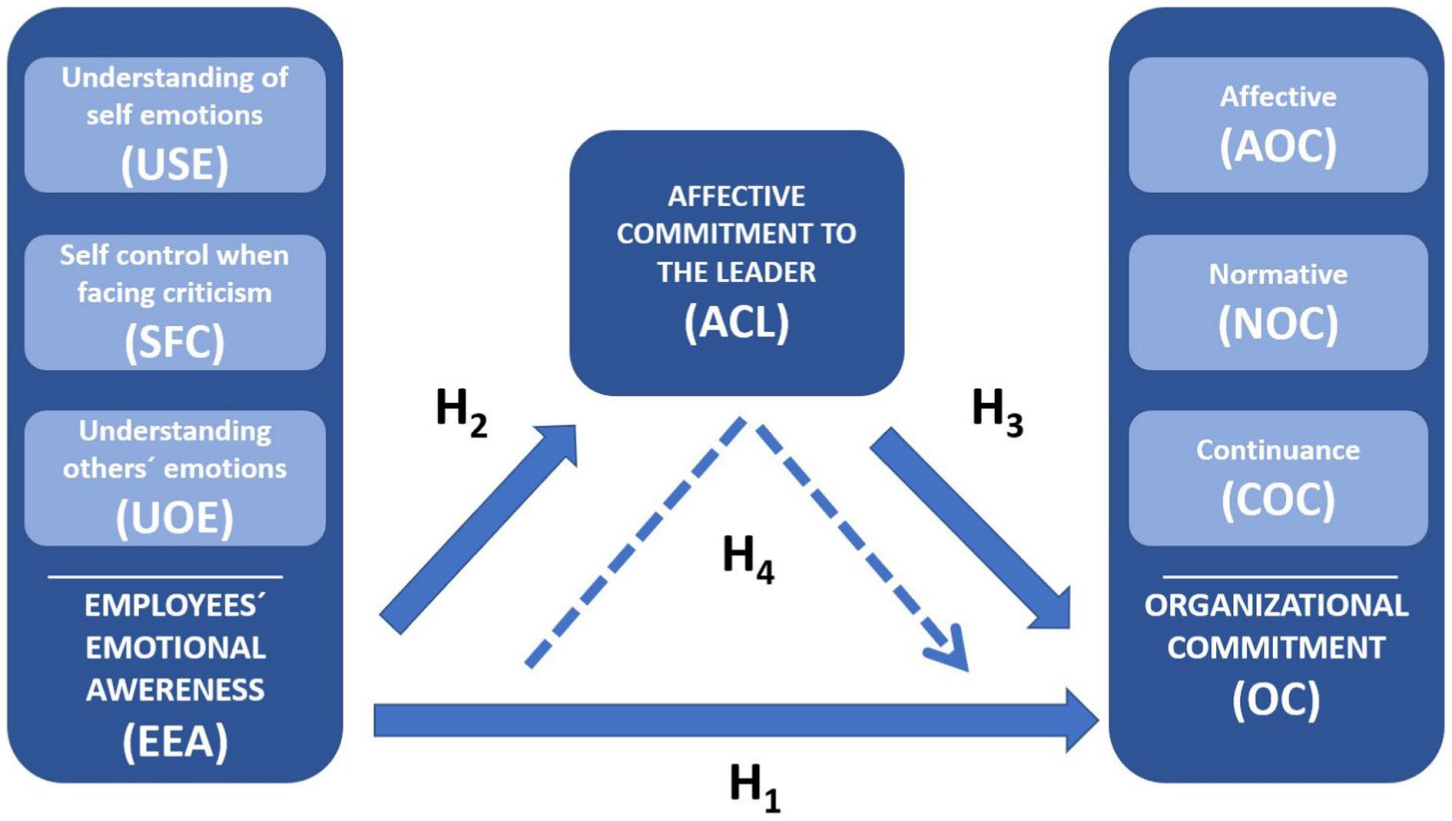
Conceptual model.

In the proposed model (see Figure 1) that lays out the hypotheses formulated above, a mediation relationship is also included (Baron and Kenny, 1986). The aim is therefore to identify whether the relationship between employees’ emotional awareness and organizational commitment is mediated by affective commitment with the leader. Many studies have suggested that the leader influence employees´ affective organizational commitment (Eisenberger et al., 2010; Stinglhamber et al., 2015; Lapointe and Vandenberghe, 2017; Benevene et al., 2018). Considering that, the leader is an important figure in the workplace, and several studies have suggested that many different types of leadership have positive influences, not only on employees positive behaviors, but also, influence the affective organizational commitment, it is expected that affective commitment to the leader play a mediating role in the relationship between employees’ emotional awareness and organizational commitment.

In view of the above, the following hypothesis was formulated:

*Hypothesis 4*: Affective commitment to the leader positively mediates the relationship between employees’ emotional awareness and organizational commitment.

## Materials and methods

### Sample

This study involved the participation of two large private organizations in Portugal, one of French nationality in the retail sector, and another multinational of Portuguese nationality in the food industry. These two companies were chosen to minimize the cultural country limitation, for the reason that both have employees of different nationalities. We have also decided to consider companies from different sectors to ensure sample diversity.

This convenience sample consisted of 403 respondents from two different sectors in Portugal: one company from the retail sector (14,000 employees) and the other company from the food industry company (3,000 employees). According to the calculation of samples for finite populations, we can consider that the sample of 403 is representative of the population of 17,000 and sample error less than 5%. It was composed of 56.1% female respondents and 43.9% male respondents, with an average age of 37 years; the minimum age of respondents was 20 years and the maximum age 65 years. Average tenure in the organization was 9 years, with the minimum tenure in the same organization being 1 year and the maximum 40 years.

### Measures

This study used a questionnaire survey as the data collection instrument. The data were subjected to statistical analysis and treatment using the *Statistical Package for Social Sciences*—*SPSS* (version 22) and *LISREL 9.2*. All measurement models were validated against the sample (with a dimension of 403 respondents) through confirmatory factor analysis (CFA). They were respecified by eliminating items with factor loading values lower than 0.5 and high modification indices (Hair et al., 2010).

The questionnaire consisted of three author scales, with answers based on a seven-point Likert scale, where “1” corresponds to “Totally Disagree” and “7” to “Totally Agree.” Employees’ Emotional Awareness was measured based on the model of Mayer and Salovey (1997) in the version adapted and validated for the Portuguese context by Rego and Fernandes (2005). It is a formative measurement model with nine items and three dimensions: Understanding self-emotions, Self-control when facing criticism, and Understanding others’ emotions. According to Hair et al. (2010), the three dimensions have indexes that support their convergent validity (respectively Understanding self-emotions: *α* = 0.875, AVE = 81%, and CR = 0.93; Self-control when facing criticism: *α* = 0.761, AVE = 58%, and CR = 0.80; and Understanding others’ emotions: *α* = 0.690, AVE = 61%, and CR = 0.82).

Regarding organizational commitment, a scale was used which was adapted and validated for the Portuguese context by Nascimento et al. (2008) based on the scale of Meyer and Allen (1997). This questionnaire consists of nine items using the three dimensions (affective, normative, and continuance) of organizational commitment, each measured by three items. These dimensions also present indexes that support their convergent validity, in line with what was stipulated by Hair et al. (2010) (Affective organizational commitment: *α* = 0.825, AVE = 68%, and CR = 0.86; Normative organizational commitment: *α* = 0.846, AVE = 69%, and CR = 0.87; and Continuance organizational commitment: *α* = 0.735, AVE = 54%, and CR = 0.78).

Finally, to measure affective commitment to the leader, the questionnaire validated by Nascimento et al. (2008) for the Portuguese context on organizational commitment was adapted for leader. This questionnaire only used the affective dimension of commitment to the leader, so three items of the scale were included in the questionnaire. It presented indexes that also support its convergent validity (Affective commitment to the leader: *α* = 0.879, AVE = 78%, and CR = 0.91).

To minimize the common method bias, the different scales have reversed items, and for the design of the questionnaire, the scales were also subjected to a random distribution of the items of which they are composed (Podsakoff et al., 2012). The variance associated with the common method bias was calculated using the common factor method (Podsakoff et al., 2003).

Following the process stipulated by Podsakoff et al. (2003), the model without the common factor is significantly different from the model with it [measured by all 21 items of the questionnaire; Δ☐^2^ = 48.37; Δdf = 21; critical value for Δ☐^2^(Δdf = 15) = 32.671 < 48.37]. On the other hand, it is also found that the inclusion of the common factor leads to a better adjustment of the model, namely in terms of RMSEA (0.068 vs. 0.059), GFI (0.906 vs. 0.928), and CFI (0.985 vs. 0.990). However, the average variance extracted (AVE) by the common factor (AVE = 20%) is lower than the reference value whereby “typical job performance measures contained an average of 22.5% method variance” (Podsakoff et al., 2003, p. 880). Considering the AVE by the common factor, despite the differences between the two models (with and without a common factor), it can be concluded that the common method biases will not have a significant influence on the estimation of the proposed model.

## Results

### Descriptive statistics

Based on the CFA, descriptive statistics of the latent variables are presented in Table 1. On analysis, it can be identified that the variable’s averages have high values, with the dimension of continuity of organizational commitment showing the lowest average value with 3.09. They have a convergent validity determined by factor loading all of the above 0.5 and AVE above 0.5. Likewise, the internal consistency and reliability are acceptable with a Construct Reliability and a Cronbach Alpha Coefficient above 0.7 in all variables (Schermelleh-Engel et al., 2003; Hair et al., 2010).

**Table 1:** Correlations between latent variables.

	M	DP	USE	SFC	UOE	A-CL	A-OC	N-OC	C-OC
USE	5.491	0.882	(0,88); [0,81]; {0,93}						
SFC	3.940	1.052	0.20**	(0,761); [0,58]; {0,80}					
UOE	4.819	0.619	0.43**	0.26**	(0,69); [0,61]; {0,82}				
A-CL	4.659	1.447	0.20**	0.25**	0.23**	(0,88); [0,78]; {0,91}			
A-OC	4.329	1.213	0.32**	0.15**	0.31**	0.41**	(0,83); [0,68]; {0,86}		
N-OC	3.938	1.380	0.27**	0.06	0.23**	0.29**	0.73**	(0,85); [0,69]; {0,87}	
C-OC	3.092	1.198	0.09	−0.15**	−0.02	−0.10*	0.08	0.10*	(0,74); [0,54]; {0,78}

USE, Understanding self-emotions; SFC, Self-control when facing criticism; UOE, Understanding others’ emotions; A-CL, affective commitment to the leader; A-OC, affective organizational commitment; N-OC, normative organizational commitment; and C-OC, continuance organizational commitment.

(), Cronbach’s Alpha Coefficient (acceptance values of 0.70); [], Average Variance Extracted (acceptance values of 0.50); and {}, Construct Reliability (acceptance values of 0.7).

*p < 0.05 and **p < 0.01.

Regarding the dimensions of emotional awareness (USE, SFC, and UOE) a significant correlation relationship was identified between Understanding self-emotions and Understanding others’ emotions (0.43). This result suggests that we could be dealing with variables with different nomenclatures that measure similar factors. In this specific case, understanding emotions is a common factor; in the dimension of Understanding self-emotions, it refers to the individual’s own emotions, and in the dimension of Understanding others’ emotions, it is about managing emotions while interactions with other people take place.

The correlation value between the normative and affective dimension of organizational commitment can also be emphasized (0.73). These data are in line with what is advocated by several authors regarding the need to reassess the commitment model, due to the fact that there may be an issue of a two-dimensional model (Meyer and Allen, 1997; Meyer et al., 2002; Meyer and Parfyonova, 2010).

Finally, a significant correlation was also found between the between affective organizational commitment and affective commitment to the leader (0.41). Once again, the affective component is common to both constructs, despite their different focis (organization and leader).

Given these results, a positive relationship was found between the three dimensions of emotional awareness (USE, SFC, and UOE) and affective organizational commitment, with statistically significant values. Regarding the relationship between the dimensions of emotional awareness and normative organizational commitment, they present positive and statistically significant results, with the exception of Self-control when facing criticism.

As for the relationship between emotional awareness and continuance organizational commitment, the only statistically significant correlation, to Self-control when facing criticism, is negative (−0.15**). Regarding the relationship between emotional awareness and affective commitment to the leader, it can be identified that all dimensions (USE, SFC, and UOE) are positively correlated and have significantly high values.

### Analysis of the structural model of the relationship between emotional awareness and organizational commitment

The model proposed has a good index for goodness of fit (Table 2).

**Table 2:** Goodness of fit index of the relation between emotional awareness and organizational commitment.

*x* ^2^	*df*	*p* value	RMSEA (<0.8)	GFI (>0.9)	IFI (>0.9)	CFI (>0.93)	*x*^2^/*df* (<0.02)	AIC (smallest value)
188.10	126.00	0.000	0.065	0.917	0.950	0.950	1.49	3,136,62

(), Acceptable Fit (Schermelleh-Engel et al., 2003; Hair et al., 2010).

**Figure 2:**
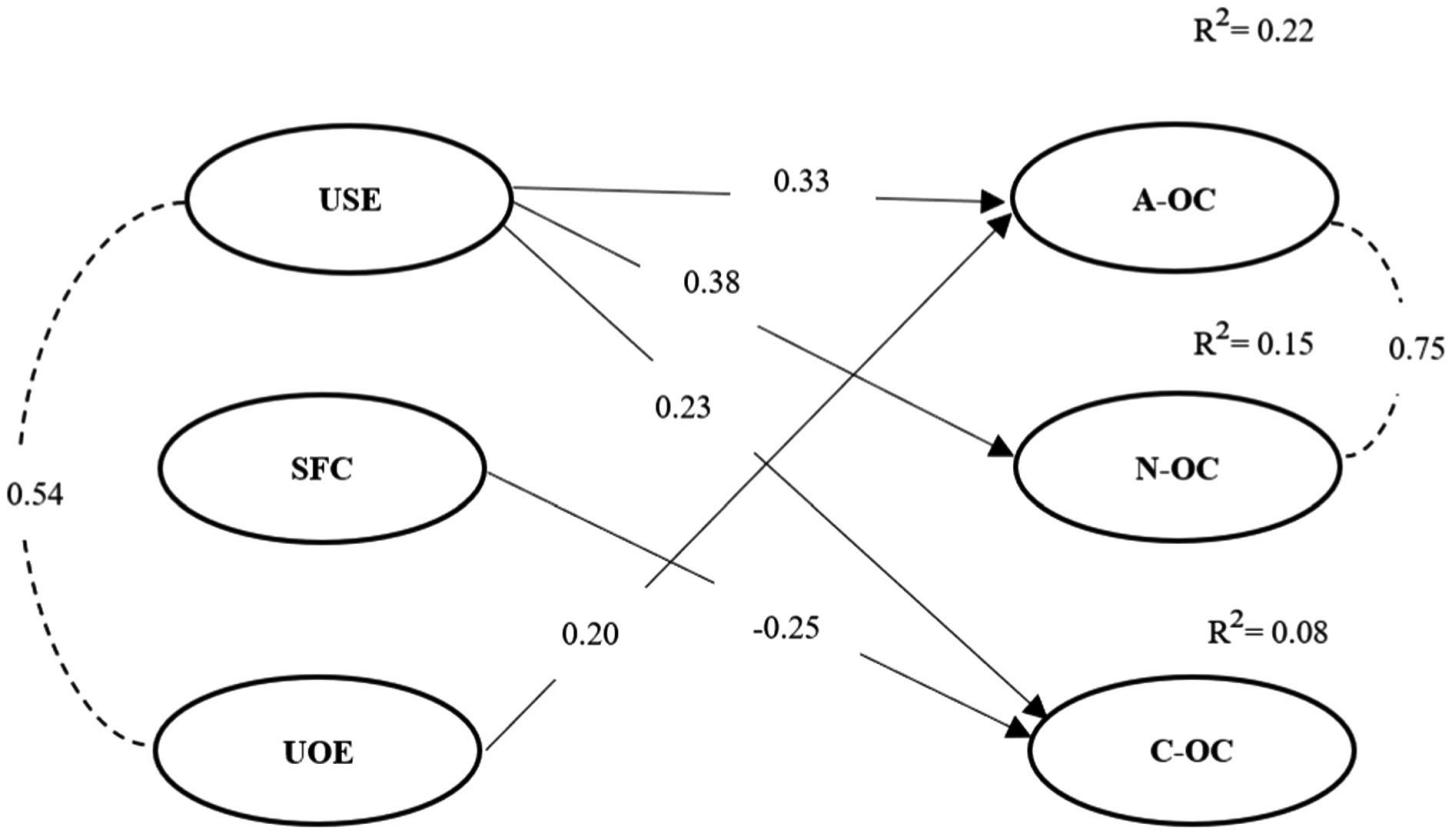
Structural model of the direct relationship between employees’ emotional awareness and organizational commitment.

Based on the results of the analysis, it was found that Understanding self-emotions has a positive relationship with the affective (0.33), normative (0.38) and continuance (0.23) dimensions of organizational commitment (Figure 2). In view of these results, we can state that H1_a_ was confirmed. Self-control when facing criticism has only a negative relationship (−0.25) with continuance organizational commitment. Thus, H1_b_ was rejected, as there was no relationship with affective organizational commitment or normative organizational commitment. In the case of Understanding others’ emotions, it only presents a (positive) relationship with affective organizational commitment (0.20). Thus, H1_c_ was partially confirmed, insofar as there was no relationship with normative and continuance organizational commitment, despite the positive relationship presented above.

The determination coefficient (*R*^2^) was also analyzed, and a relationship of influence of the dimensions of emotional awareness regarding the three dimensions of organizational commitment (affective, normative, and continuance) was identified. We found that Understanding self-emotions and Understanding others’ emotions explain 22% of affective organizational commitment. It is also noteworthy that Understanding self-emotions explains 15% of normative organizational commitment. In the case of continuance organizational commitment, 8% is explained by its positive relationship with Understanding self-emotions, and by the negative relationship with Self-control when facing criticism.

### Analysis of the effect of mediation of affective commitment to the leader

After analyzing the structural relationships and testing specific hypotheses between emotional awareness and organizational commitment, according to the methodology established by Baron and Kenny (1986), MacKinnon et al. (2007), and Hair et al. (2010) the mediating variable was introduced in the final model of direct structural relationships, in this case, affective commitment to the leader. The model obtained shows good goodness of fit index (Table 3).

**Table 3:** Goodness of fit index of the relation between employees’ emotional awareness, affective commitment to the leader, and organizational commitment.

*x^2^*	*df*	*p* value	RMSEA (<0.8)	GFI (>0.9)	IFI (>0.9)	CFI (>0.93)	*x*^2^/*df* (<0.02)	AIC (smallest value)
262.82	175.00	0.000	0.068	0.901	0.984	0.984	1.501	3,325.57

(), Acceptable Fit (Schermelleh-Engel et al., 2003; Hair et al., 2010).

**Figure 3:**
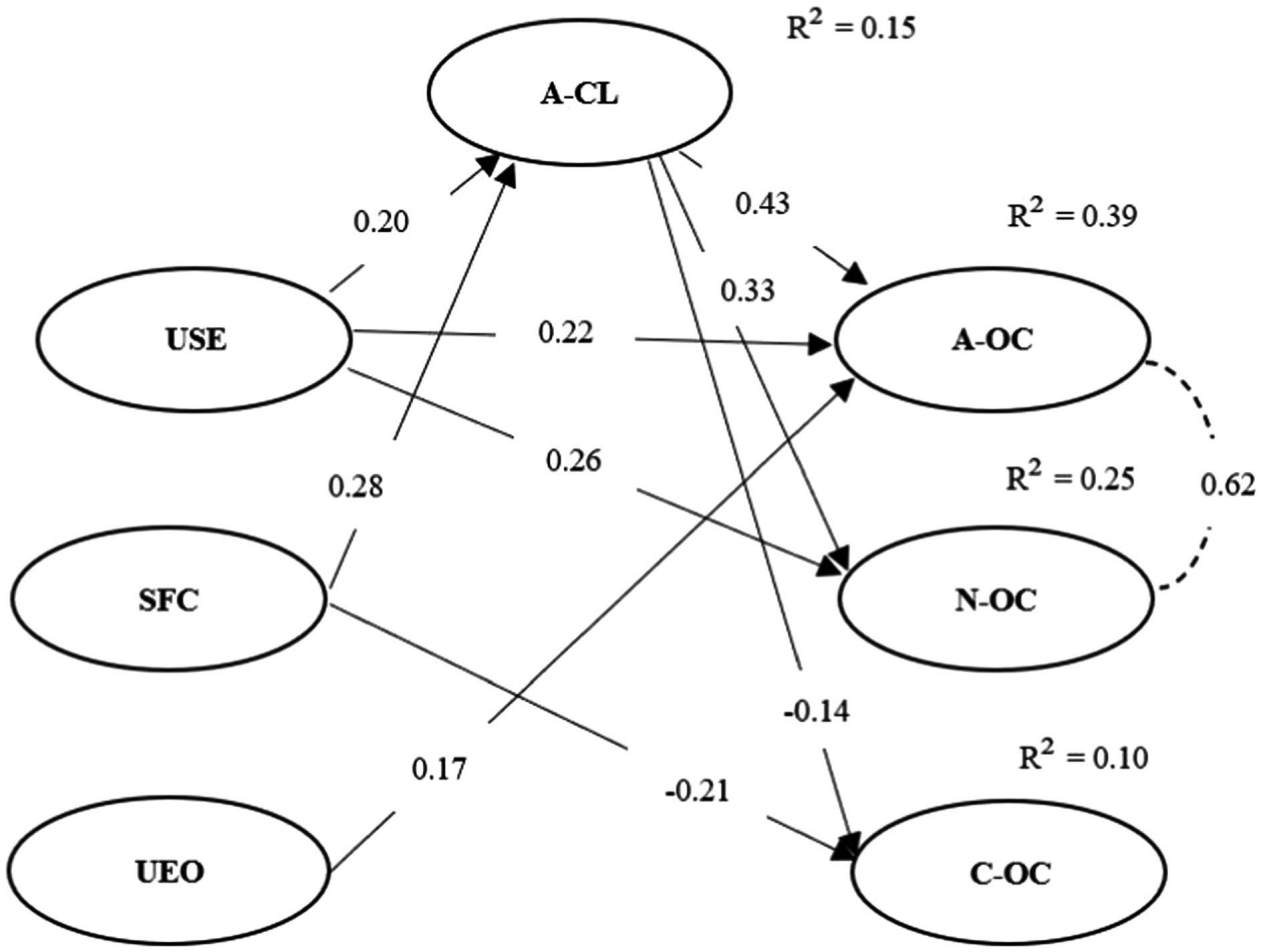
Diagram of the final model of the relationship between employees’ emotional awareness, affective commitment to the leader, and organizational commitment.

Regarding the relationship between employees’ emotional awareness and affective commitment to the leader, the analysis of these relationships started from the relationships in H1, that is, from the direct relationship between emotional awareness and organizational commitment. Thus, it was identified that, in the presence of affective commitment to the leader, relationships between the dimensions of Emotional awareness and organizational commitment changed (see Figure 3). Understanding self-emotions ceased to be positively related to continuance organizational commitment, and the strength of the relationships between the other dimensions of emotional awareness and organizational commitment decreased.

Regarding the relationship between the dimensions of emotional awareness and affective commitment to the leader, there was a positive relationship with Understanding self-emotions (0.20) and with Self-control when facing criticism (0.28). Thus H2_a_ and H2_b_ were confirmed. However, H2_c_ was rejected, as Understanding others’ emotions did not reveal any relationship with affective commitment to the leader.

As for the relationship between affective commitment to the leader and organizational commitment, a positive relationship was identified with the affective (0.43) and normative (0.33) dimensions and a negative association with continuance (−0.14). So these results partially support the H3.

On the other hand, there was a negative relationship with the continuance dimension of organizational commitment. These results are not aligned with the established theoretical framework. As a result of including the affective commitment to the leader in the model, we identified that Understanding Self-Emotions and Self-Control when facing criticism explained 15% of affective commitment to the leader. There is also an increase in the coefficient of determination in the different dimensions of organizational commitment, where 39% of affective organizational commitment is explained by the relationships of the final model, 25% of normative organizational commitment, and 10% of continuance organizational commitment. Thus, the data suggest that affective commitment to the leader plays a mediating role in organizational commitment.

### Mackinon’s *Z* test of the mediation of affective commitment to the leader between employees’ emotional awareness and organizational commitment

The final structural model reflects the mediation effect of affective commitment to the leader in the relationship between the dimensions of emotional awareness and organizational commitment. It identified that mediation conditions exist, as both indirect effects are statistically significant (Baron and Kenny, 1986; MacKinnon et al., 2007; Hair et al., 2010). In view of these results, the significance of indirect effects was tested using Mackinnon’s *Z*.

General Hypothesis 4 posits the possibility of mediation existing of affective commitment to the leader in the relationship between employees’ emotional awareness and organizational commitment. Thus, the final model identified the mediation relationships (Table 4).

**Table 4:** Mediation relationships of affective commitment to the leader in the relation between employees’ emotional awareness and organizational commitment.

Mediation relationships	*Z*’(*Z*’ ≥ 0.97)	Conclusion
Mediation of A-CL between USE and A-OC	*Z*’ = 2.856	Not rejected
Mediation of A-CL between USE and N-OC	*Z*’ = 2.688	Not rejected
Mediation of A-CL between USE and C-OC	*Z*’ = -1.710	Not rejected
Mediation of A-CL between USE and C-OC	*Z*’ = -1.816	Not rejected

Given the above, the results confirm the mediation relationship as presented in Hypothesis 4. Thus, the data indicate that affective commitment to the leader mediates the relationship between Understanding self-emotions and the affective, normative, and continuance dimensions of the organizational commitment, as well as mediating the negative relationship between Self-control when facing criticism and the continuance variable of organizational commitment.

## Discussion

Research from different authors have suggested carrying out studies that allow identification of antecedents, different focis of commitment, causes, effects, changes over time, directionality, and profiles, and motivating to a better understanding of commitment and the behavioral phenomena that it involves (Bergman et al., 2013; Meyer et al., 2015; Eisenberger et al., 2019; Klein et al., 2020; van Rossenberg et al., 2022). A contribution that reconciles the academic and practical perspectives has also been sought.

The results of this study emerged from an analysis of the antecedence relationship of the emotional awareness dimensions in relation to each dimension of organizational commitment. The results suggested that understanding self-emotions influences positively the three dimensions of organizational commitment (affective, normative, and continuance). Thus, it can be said that a greater degree of Understanding self-emotions, which is, being able to interpret and manage one’s own emotions, promotes the conscious development of bonds that lead to organizational commitment. In this specific case, it can also be mentioned that this ability, despite its positive influence on the three dimensions of organizational commitment, has higher relationship values in the affective and normative dimensions (social exchange), and lower with the continuance dimension (financial exchange). This reveals that the conscious understanding of one’s own emotions favors the development of bonds of commitment, preferably affective, and of moral obligation toward the organization.

When affective commitment to the leader is included in the model of the direct relationship between emotional awareness and organizational commitment, a significantly positive relationship between Understanding self-emotions and affective commitment with the leader was found, but the relationship between Understanding self-emotions and continuance organizational commitment also disappeared. This result suggests that the strength of the affective commitment bond to the leader reduces the tendency for continuance organizational commitment.

Regarding to Understanding others’ emotions, only its positive influence on affective organizational commitment was identified. This result is in line with the established theoretical framework, which states that a greater ability to consciously interpret others’ emotions enables individuals to develop more satisfying and empathic interpersonal relationships. Thus, a higher level of Understanding others’ emotions enhances the creation of affective or relational bonds; this will have consequences for the perception of the organizational context, and for the way in which employees analyze and assess the organization as a whole, developing affective organizational commitment.

Self-control when facing criticism showed a negative relationship with continuance organizational commitment. The theoretical framework argues that an employee’s inability to constructively control emotions in feedback contexts may have an implicit rationale of fear, losing one’s job, or having a lack of other professional options (Smith et al., 2018). Therefore, this context can influence the development of organizational continuance commitment. Conversely, the greater the ability to accept criticism in a constructive and positive way and use it for one’s own development, the lesser the tendency to link the context to the development of continuance organizational commitment.

The results also suggest the possibility that Self-control when facing criticism leverages affective commitment toward a personal commitment foci, and consequently a negative trend toward continuance organizational commitment. It should be recalled that in the final model (Figure 3), Self-control when facing criticism started by showing a positive relationship with affective commitment with the leader. It can be assumed that managing one’s emotions and a positive attitude toward feedback may contribute to the positive relationship between employee and supervisor, strengthening affective commitment to the leader and decreasing continuance organizational commitment.

Thus, the greater the employees ‘emotional awareness, the greater their predisposition to affective commitment to the leader and the organization. The positive relationship of emotional awareness with two focis of commitment is linked to the possibility of having an antecedent of other focis of commitment.

Some authors such as Klein et al. (2009) or Meyer et al. (2012) argue that the affective dimension of commitment is actually the bond with the greatest strength. The results of this study corroborate this view, because both in relation to the organization and in relation to the leader, the coefficients of determination of the affective dimensions showed higher values than the continuance dimension. In view of this, it is also worth noting that affective commitment to the leader had a significant positive influence on affective organizational commitment and normative organizational commitment.

Recent studies which focused on commitment profiles, also suggested a strong correlation between affective and normative variables. The proposal is that this may constitute a moral duty profile, in which the employees are committed to the organization because they like it, but also because they feel a duty to contribute to organizational goals (Meyer and Parfyonova, 2010; Meyer et al., 2012).

Regarding the relationship between affective commitment to the leader and continuance organizational commitment, the relationship was found to be negative. We can therefore assume that, in the presence of an affective bond to the leader, the continuance bond not only loses relevance but is also inverse, i.e., negative. This result highlights the influence of commitment to the leader in forming organizational commitment.

Therefore, in line with what has also been argued by other authors, the results reveal the mediating role of affective commitment to the leader regarding the strength of the affective bond (Klein et al., 2009; Meyer et al., 2012; Klein, 2013).

The final model thus suggests that affective commitment to the leader has a mediating role in organizational commitment, specifically in the relationships between Understanding self-emotions and the three dimensions of organizational commitment and in the relationship between Self-control when facing criticism and continuance organizational commitment (see Figure 2).

### Theoretical contributions

The aim of this research was to contribute to the area of studies on commitment, reconciling two lines of research into commitment, one focused on identifying antecedent variables, and the other addressing the relationship between two focis of commitment in the workplace: the leader and the organization.

An analysis of the structural relationships was chosen in which three independent variables that reflect emotional awareness were established. An antecedent perspective was taken, with an approach to the multidimensionality of organizational commitment (affective, normative, and continuance), and the unidimensionality of affective commitment to the leader, from a relationship mediator perspective.

Thus, in view of the antecedent analysis regarding the two focis of commitment, the present study suggests that employees’ emotional awareness, in particular Understanding self-emotions, Self-control facing criticism and Understanding others’ emotions, influence the process of commitment to the leader and the organization, according with Affective Events Theory. The study also reveals that these relationships change according to the presence or absence of affective commitment to the leader, supporting the social exchange theory, more specifically the Leader Member Exchange Theory.

Based on the results, it can be stated that employees’ emotional awareness, particularly Understanding self-emotions, can engender a greater predisposition to organizational commitment in its three dimensions: affective, normative, and continuance. Moreover, Understanding others’ emotions positively influences affective organizational commitment. These results suggest that, according to Meyer and Allen’s (1997) model, employees’ emotional awareness may be a distant antecedent of commitment, as it is a personal characteristic of the employee.

Thus, the way in which the internal process of analyzing emotions is carried out, which may be a developed competence, is inherent to the individual around his experience with the external context. This dynamic is also subject to the experiences that individuals experience over time, carrying out different tasks in different organizational contexts. Individuals’ behavior patterns are subject to the need to be permanently updated or renewed, depending on the experiences and needs of each individual’s adaptive process, which is dynamic and continuous throughout life (Rimé, 2015; Smith et al., 2018).

For Rimé (2015) this process is complex, internal and in some cases time-consuming, which do not happen continually in moments of interaction with what is external to the individual. According to this author, the way in which this analysis process is undertaken internally may interfere with the way in which the individual overcomes obstacles, which may also impact an individual’s commitment process.

A mediating role of affective commitment to the leader was also found, with the relationship between the dimensions of emotional awareness and organizational commitment changing. This dynamic showed that affective commitment to the leader inhibits the relationship between Understanding self-emotions and continuance organizational commitment. This result suggests that the affective bond with the leader is inverse to the continuance organizational commitment. Also, Self-control when facing criticism is shown to have a negative relationship with the continuance organizational commitment in its positive relationship with the affective commitment to the leader, which corroborates the previous interpretation of the results. Along these lines, regarding the relationship and directionality between the two commitment focis, we highlight the positive relationship between the affective commitment to the leader and affective and normative organizational commitment, and the negative relationship with continuance organizational commitment. This result again highlights the force of the affective bond in the commitment process. It should be noted that Klein et al. (2020) found that the continuance bond was more frequently reported regarding the organization than other focis. The result of our study corroborates this conclusion, from the perspective that the tendency of continuance commitment will decrease in the presence of an affective commitment to another target.

Finally, the positive correlation between the affective and normative dimension of organizational commitment should be highlighted. This result, once again, suggests the possibility that we there is a need to adjust the model, which could potentially be two-dimensional. Another interpretation, in line with studies of latent profiles, this result may suggest that what we are witnessing is a profile of moral duty (Meyer and Parfyonova, 2010; Meyer et al., 2012, 2015).

### Practical implications

Commitment has been considered a significant subject in the strategy of organizations which aim to be competitive and develop a culture of high performance (Beer et al., 2015; Culibrk et al., 2018; Bak, 2020; Lee et al., 2020).

The challenges that commitment presents for management, especially people management, are fundamentally related to the difficulty of understanding what it is that enhances employee commitment; what the commitment bonds are; as well as determining the set of focis is to which employees commit themselves in the organizational context (Beer et al., 2015; van Rossenberg et al., 2022).

Thus, this work identified that employees’ emotional awareness has positive implications in their process of organizational commitment. It can be said that the emotional maturity of employees, that is, their ability to manage emotions more consciously, enhances their ability to satisfactorily understand and manages their daily lives, even in stressful situations (Mayer and Salovey, 1997; Smith et al., 2018; Zia et al., 2018; Helvac and Yilmaz, 2020). According to the results of this study, understanding and conscious management of one’s own emotions lead individuals to be more aware of what they want and what they seek as people and professionals. This condition enhances the creation of commitment bonds, in which the employee understands why he likes the organization, what makes him feel grateful to the organization and how he values instrumental aspects (career, salary, etc.).

The ability to understand others’ emotions also has positive implications for affective organizational commitment, since this ability to manage and adapt one’s emotional state according to those with whom one interacts tends to provide healthier and more satisfying relationships. This leads to a more positive perception of the organization and consequently leads to bonds of an affective nature.

It can thus be concluded that the development and emotional training of employees (from the basis until the top organizational positions) can be considered a measure that encourages the potential creation of commitment bonds in its different dimensions (affective, normative, and continuance).

It is common for organizations to opt for measures of an instrumental nature as a way to foster commitment. Investing in an attractive remuneration package may seem relatively less complex than implementing measures of a more abstract nature, such as affection, gratitude, or loyalty. However, the results show that a focus on measures for continuance organizational commitment is not necessarily more effective, whereby other organizations merely need to be willing to match or exceed the instrumental offer.

The present study highlighted the strength of the affective and normative bond in relation to the continuance bond (instrumental). On the one hand, affective and normative bonds were identified as showing stronger results in the relationship between emotional awareness and organizational commitment. On the other hand, in the presence of an affective commitment bond to the leader, there was a significant weakening of the continuance organizational commitment. These results suggest two relevant aspects; (i) the affective bond is potentially stronger and enhances commitment regarding other focis; and (ii) the leader is a commitment target with strong implications for affective and normative organizational commitment. Thus, the suggestion is that organizations should develop a culture of ethical leadership, where the leader also assumes a coaching role, contributing feedback to promote employees’ development (Eisenberger et al., 2010; Bak, 2020; Saeed et al., 2022).

It should also be mentioned that development of affective commitment to the leader and positive feedback for employees significantly influences innovative work behaviors and organizational commitment (Bak, 2020). Given that, the present study suggests that a greater capacity for self-control when facing criticism favors affective commitment with the leader, developing employees’ emotional awareness is suggested to enhance organizational commitment and affective commitment with the leader.

In short, a need can be identified to develop employees’ emotional awareness and evolution, but also to reinforce the importance of leaders adopting a leadership style that enhances the employees’ commitment to themselves and to the organization.

### Limitations and future directions

This study did not identify a leadership style, nor did it measure the leaders’ emotional awareness, namely from the perspective of his subordinates. This analysis could contribute to parallel readings regarding the results. This fact is not only a limitation, but also a recommendation for future studies.

The controversy that exists around the definition, measurement, and differences between emotional awareness and emotional intelligence may also be taken as a limitation, such that other scales may provide different results.

In the line of research used to carry out this work, it is also suggested to carry out studies that use other antecedent variables, such as employees’ values and organizational values. The replication of this study using other scales, as well as other samples of greater size and diversity may identify similarities and differences, and contribute to new confirmations or conclusions. Undertaking studies with other objects of commitment present in the workplace is also suggested in order to identify their relationships and directions. In this vein, we also suggest to conduct multilevel studies to identify the influence of commitment between different hierarchical levels.

Longitudinal studies would also be extremely relevant for the study and understanding of commitment in order to identify the evolutions and dynamics over time of the different types, levels, and focis of commitment. The combination of this type of studies with the line of research into latent profiles would potentially reveal relevant suggestions for the study and evolution of the understanding of this construct.

## Conclusion

The context to which organizations have been exposed has forced permanent organizational change. From changes in the labor market to technological transformation or even to socio-economic conditions, organizations have faced enormous challenges and uncertainties. The need to become more competitive, with a greater capacity for innovation that allows them to mark themselves as distinct in the market has led organizations to position their human capital as an important business driver (Ulrich, 2013; Strack et al., 2014; Beer et al., 2015; Markoulli et al., 2017; Culibrk et al., 2018). In this context, commitment is seen as a topic of great strategic importance, as it contributes not only to retaining talent, but also to enhancing better performance (Culibrk et al., 2018; Bak, 2020; Klein et al., 2020; Lee et al., 2020). It is thus urgent to provide organizations with knowledge that contributes to strengthening their people management strategies, particularly within the scope of employee commitment, creating conditions for employees to deal with uncertainties and organizational changes and develop interests and commitment bonds in common with leadership and organization (Morrow, 2011; Bergman et al., 2013; Klein, 2013; Meyer et al., 2015).

In this study, the importance of employees’ emotional awareness and its influence on organizational commitment and commitment to the leader was evident. It can be posited that employees’ emotional maturity, namely identifying, perceiving, and learning from their emotions in the organizational context, provides more favorable conditions for commitment, considering different focis. The relevance of the affective bond with two commitment focis of the study was also noticeable, as it negatively impacted the instrumental bond of continuity organizational commitment in the presence of the affective commitment with the leader.

## Data availability statement

The raw data supporting the conclusions of this article will be made available by the authors, without undue reservation.

## Author contributions

All authors listed have made a substantial, direct, and intellectual contribution to the work and approved it for publication.

## Funding

This research has been partially funded by the Regional Government of Extremadura (Junta de Extremadura) and the European Union (European Regional Development Fund - A way of making Europe), supporting Research Groups (SEJO21 – GR21078) of the University of Extremadura.

## Conflict of interest

The authors declare that the research was conducted in the absence of any commercial or financial relationships that could be construed as a potential conflict of interest.

## Publisher’s note

All claims expressed in this article are solely those of the authors and do not necessarily represent those of their affiliated organizations, or those of the publisher, the editors and the reviewers. Any product that may be evaluated in this article, or claim that may be made by its manufacturer, is not guaranteed or endorsed by the publisher.
